# Intra-arterial chemotherapy for retinoblastoma: Our first three-and-a-half years’ experience in Malaysia

**DOI:** 10.1371/journal.pone.0232249

**Published:** 2020-05-01

**Authors:** Chee Chung Liu, Adzleen Mohmood, Norhafizah Hamzah, Jia Him Lau, Nurliza Khaliddin, Jamalia Rahmat

**Affiliations:** 1 University Malaya Eye Research Centre, University of Malaya, Kuala Lumpur, Malaysia; 2 Department of Ophthalmology, Hospital Kuala Lumpur, Kuala Lumpur, Malaysia; 3 Department of Ophthalmology, University Kebangsaan Malaysia, Bangi, Selangor, Malaysia; 4 Department of Radiology, Hospital Kuala Lumpur, Kuala Lumpur, Malaysia; Massachusetts Eye & Ear Infirmary, Harvard Medical School, UNITED STATES

## Abstract

**Aim:**

To report our first three-and-a-half years’ experience with intra-arterial chemotherapy (IAC) in managing retinoblastoma (RB).

**Methods:**

Single institution, retrospective, interventional case series of 14 retinoblastoma patients managed with IAC from December 2014 to June 2018. Demographics were described. Outcomes measures were tumor response, treatment complications and globe salvage.

**Results:**

Subjects’ mean age at the first administration of IAC was 31.4 months. 57.1% of the eyes were Group D and E retinoblastoma, while 79% were bilateral disease. 93% of the eyes were as secondary treatment. Of 32 IAC cannulations performed, 23 (71.8%) were successful and received chemotherapy drug melphalan. Each eye received a mean of 1.8 (range 1–4) IAC injections. 53% of the eyes showed regression post treatment. After a mean follow up period of 19 months, globe salvage rate was 38%. Most of the adverse effects experienced were localized and transient.

**Conclusion:**

IAC has provided an added recourse in the armamentarium of retinoblastoma treatment in our center. IAC treatment is a viable alternative in the treatment of retinoblastoma to salvage globe, for eyes that would conventionally require enucleation especially in bilateral disease.

## Introduction

Retinoblastoma (RB) is the most common intraocular malignancy in children. This aggressive cancer is fatal if left untreated, but early diagnosis and treatment advancement have made RB a highly curable disease. Conventionally, the main aim of retinoblastoma management is focused on the patient's survival, with secondary attempt to salvage the globe and vision. However, the current trends of treatment also focused on maximizing ocular salvage while conserving vision and limiting side effects.

Over the last decade, intra-arterial chemotherapy (IAC) has gained popularity worldwide as a promising therapy in RB. It provides a high success rate of globe salvage and tumor control, with a good safety profile. [1, 2] In IAC, the chemotherapeutic drugs are precisely injected into the ophthalmic artery, minimizing its systemic toxicity. IAC has since allowed many eyes that previously would have required enucleation, to be salvaged.[3] World-wide, certain RB referral centers now consider IAC as the first-line treatment for RB, or as second-line therapy after failure of intravenous chemotherapy (IVC)and other interventions. Literatures on IAC treatment in retinoblastoma are limited, especially in South East Asia region. Most of the literatures were retrospective, single-arm case series. [4]

Malaysia is a developing country where most retinoblastoma cases present late with advanced disease. [5] Hospital Kuala Lumpur is the largest tertiary hospital in Malaysia and the national referral center for retinoblastoma cases. In 2017 and 2018, we managed a total of 44 new retinoblastoma cases, averaged around 20 cases each year. Our first IAC was performed in 2014. In this study, we aim to report our first three and half years’ experience with retinoblastoma managed with intra-arterial chemotherapy (IAC).

## Materials and methods

This was a single institution, retrospective, interventional case series of 14 retinoblastoma patients managed with IAC over three and a half years period (December 2014 until June 2018).

Medical records and fundus photography (Retcam^®^ imaging) of all patients treated with IAC at Hospital Kuala Lumpur from 1 December 2014 to 30 June 2018 were reviewed. The recorded data included patient’s demographics, characteristics of each RB eye, tumor classification according to International Classification of Retinoblastoma[6], follow-up duration, details of prior treatment received, indication of IAC, age of patients at first administration of IAC, number of cannulation attempts and IAC administrations, routes of cannulation, technical success rate as well as adverse events during procedure. Indication of IAC was categorized into primary (treatment naïve tumor) and secondary (which included persistent tumor and recurrent tumor). Persistence of tumor includes non-regressing main tumor mass, the presence of vitreous seeds or subretinal seeds and progressive disease despite maximum tolerable treatment. Recurrent tumors showed increased tumor size or re-appearance of seeds after an initial regression with treatment.

Outcome measures were treatment complications, tumor response and globe salvage. Tumor response was categorized into regression (complete or partial) [7]; no response/persistent (similar degree of residual viable tumor/seedings); and progression (tumor advancement in size and seedings), as per clinical findings on the next examination under anesthesia (EUA). Globe salvage was defined as eyes that avoided enucleation.

This study was conducted according to the principles of the Declaration of Helsinki. Subjects’ case notes were reviewed retrospectively, and their data were kept anonymous. This study was registered at the National Medical Research Registry/ Malaysia Research Ethical Committee (NMRR ID 45797). The treatment options, rational and risks of IAC were explained in detail to patients’ parents/ guardian. Written informed consents were obtained from patients’ parents/ guardians prior to commencement of treatment. Meanwhile, the approval for publication was obtained from the Director General of Health Malaysia.

All patients with retinoblastoma who underwent IAC procedure within the study period, either as a secondary treatment or primary treatment were included in the study. Subjects receiving IAC as secondary treatment had received intravenous chemotherapy (IVC) of Carboplatin 600mg /m2, Etoposide 300mg /m2 and Vincristine 1.5mg /m2 for 6–9 cycles at 3–4 weeks intervals as a prior treatment, in combination with various focal treatments such as laser therapy, cryotherapy, or intravitreal chemotherapy as per indicated. Subjects who received IAC as primary treatment were treatment naïve. In bilateral disease, an eye was chosen for IAC if the eye belonged to a worse ICRB Group or if the fellow eye had been enucleated.

Eyes with extraocular retinoblastoma (tumor extension into optic nerve or extra scleral structures/ adjacent compartments) were excluded from the study. Eyes with attempted IAC but failed cannulation were excluded from the analysis of outcome measures.

### IAC technique

IAC is a challenging procedure that requires fine skills, experience and utmost patience. All our IAC procedures were performed by the interventional radiologist. Our technique was similar to that of other studies. [8, 9] The procedure was performed under general anesthesia in a sterile environment. Groin puncture site was cleaned and draped. Under ultrasound guidance, the femoral artery was catheterized using 21G needle, and a 4-Fr pediatric catheter with guide wire was guided into the ipsilateral internal carotid artery (ICA). The arterial vasculature was visualized under roadmap guidance. The routes of cannulation were either from the internal carotid artery (ICA) to the ophthalmic artery (OA) (23 attempts, 71.8%) or via the external carotid artery (ECA),the middle meningeal artery (MMA) and then to the OA (9 attempts, 28.1%), according to the anatomical variance of each patient.

**Fig 1:**
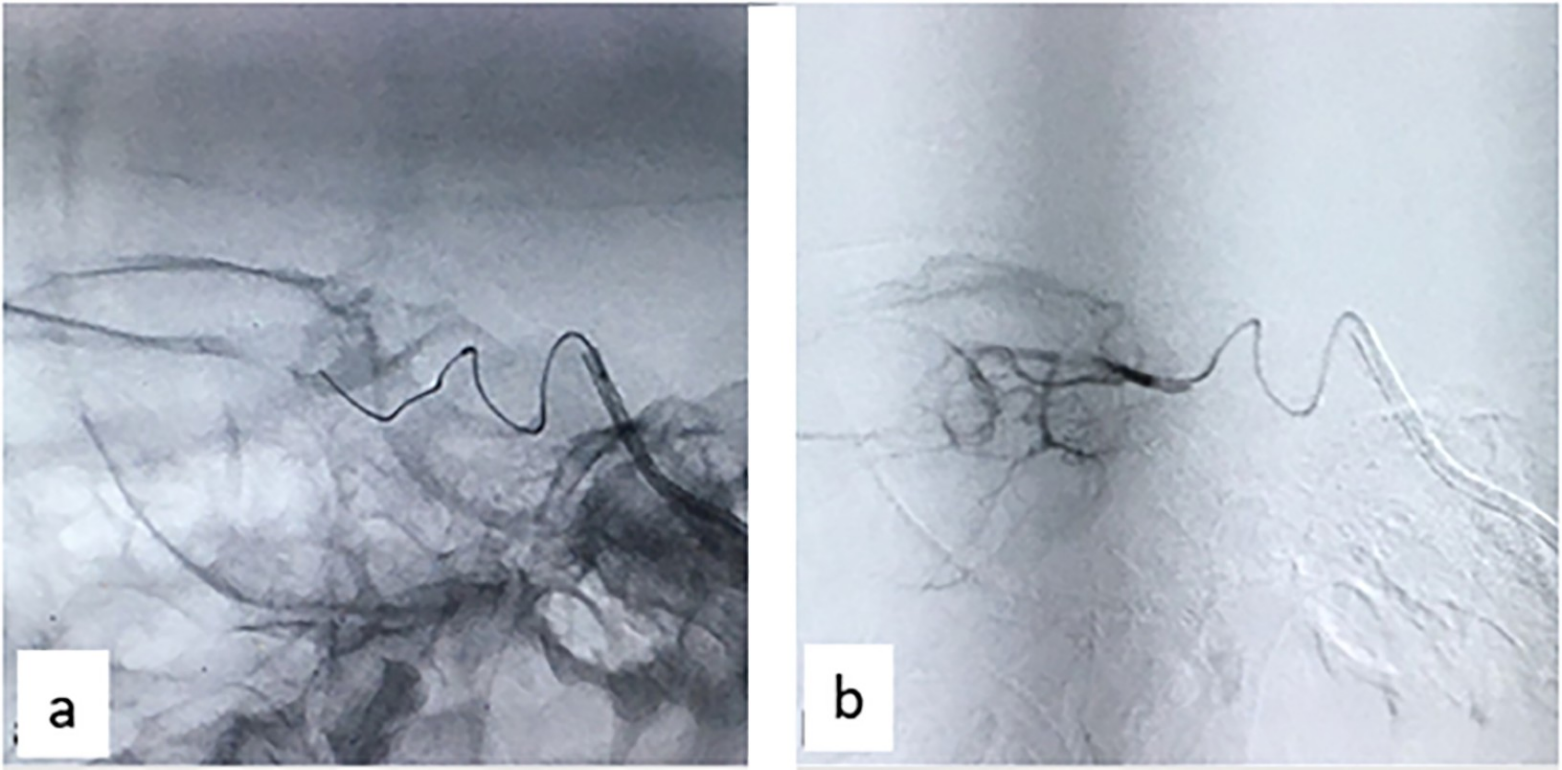
Cannulation of ophthalmic artery. (a) Ophthalmic artery cannulated using micro-catheter and guidewire under roadmap guidance. (b) Injection of chemotherapy drug Melphalan into the ophthalmic artery.

The ostium of the ophthalmic artery (OA) was then super-selectively catheterized using a microcatheter. Microcatheter's position and stability were checked and flow or reflux was assessed before chemotherapy drug was injected into the OA. We used only melphalan as the chemotherapy drug during the study period. The dose was 3 mg for patients ≤2 years and 5 mg for patients >2 years [8, 10]. Melphalan was diluted in 30 ml saline and manually injected in a slow pulsatile manner over 30 minutes (Fig 1). At the end of the infusion, the microcatheter was slowly withdrawn and roadmap angiography was again used to verify cerebral arterial flow. Lastly, the guidewire and introducer catheters were removed, and the femoral artery was compressed for 10 min to secure hemostasis at the puncture site.

After the IAC treatment, complete ocular examination under general anesthesia (EUA) was performed every 3-weekly. At each examination, the tumor response was classified as regression, no response or progressive. All fundus images were recorded using Retcam at each EUA. Tumor photos were compared to previous examination photos and classified accordingly after agreement by at least two pediatric ophthalmology consultants. The indication for a repeat treatment with IAC was persistent or no response to previous IAC. There were eyes indicated for a repeated treatment but did not receive it either due to the non-availability of melphalan at certain points of time or parent’s disagreement for repeat IAC. Eyes with disease progression despite IAC treatment were enucleated.

## Results

There were 14 eyes in 14 patients with retinoblastoma treated with IAC within the three and a half years. The demographics and features of the eyes are presented in Table 1.

**Table 1:** Demographics and features of eyes and IAC.

Features at the time of IAC	Number	%
**n = 14**		
**Mean age (in months), (median, range)**	31.4, (31, 8–77)	
**Mean follow up post IAC (in months), (median, range)**	19 (17, 5–38.5)	
**Sex**		
** Male**	8	57
** Female**	6	43
**Laterality**		
** Unilateral**	3	21
** Bilateral**	11	79
**Eyes underwent treatment**		
** Right**	6	43
** Left**	8	57
**Initial ICRB Classification by Eyes**		
** Group A**	0	0
** Group B**	3	21.4
** Group C**	3	21.4
** Group D**	7	50.0
** Group E**	1	7.1
**Previous treatment strategies by eyes**		
** IV Chemo + Laser**	1	7
** IV Chemo + Laser + Cryotherapy**	2	14
** IV Chemo + Laser + Cryotherapy + IVT chemo**	7	50
** IV Chemo + IVT chemo**	3	21
** Nil**	1	7
**Indication for IAC**		
** Persistent tumor**	8	57
** Recurrence of tumor**	5	36
** Primary**	1	7
**IAC procedure attempt**	32	
** Successful cannulation**	23	71.9
** Failed**	9	28.1
** Dissection of OA**	1	3.1
** Small/narrow OA**	6	18.8
** OA occlusion (thrombosis)**	1	3.1
** Cardiorespiratory instability**	1	3.1
**Number of successful IAC cycles**	23	
** Range**	0–4	
** Mean**	1.64	
**Treatment response by eyes**		
** Regression**	7	53
** Complete**	5	
** Partial**	2	
** No Response/ persistent**	3	23
** Progression**	3	23

IAC, intra-arterial chemotherapy; IV, intravenous; ICRB, International Classification of Retinoblastoma; OA, ophthalmic artery; IVT, intravitreal injection

IAC was delivered as a secondary therapy in 13 out of 14 eyes (93%) after previous failed treatments, and as primary therapy in one eye (7%). The local therapies received were laser phototherapy (10/14 eyes, 71%), cryotherapy (9/14 eyes, 64%) and intravitreal melphalan (10/14 eyes, 71%). Seven eyes (50%) received a combination of four treatment modalities namely intravenous chemotherapy, cryotherapy, laser therapy, and intravitreal chemotherapy.

A total of 32 cannulation attempts were performed (mean of 2.3 attempts each eye). Of these, 23 attempts were successful (in 13 eyes) with a technical success rate of 71.8%. Failure in cannulation was mostly caused by a small or narrow ophthalmic artery (6 cases, 66.7%). Only unilateral IAC was performed in this study (no eye received bilateral IAC). The mean follow-up period post IAC was 19 months (ranged 5–38.5 months). Summaries of each case are shown in Table 2.

**Table 2:** Subject demographics, tumor features, treatment, outcome measures and follow-up of IAC treatment in 14 cases.

Demographics			Tumor features			Treatment of IAC				Outcome measures			
Subject (Eye)	Age (months)/ Gender	Age Dx/ Tx (months)	Laterality	ICRB	Fellow Eye (ICRB or Normal)	Prior treatment	Line of treatment	Indication	Attempts; successful	Complication	Tumor Response*	Globe salvage	Follow up (months)
**1**	4/M	4/8	Bi	E	B	IVC, TT, CT, IVT	Secondary	Persistent	1; 1	Nil	Complete regression	Yes	32
**2**	7/F	7/29	Uni	D	Nl	IVC, TT, IVT	Secondary	Recurrence	1; 1	Nil	Progression	No	19
**3**	1.5/F	1.5/77	Bi	B	B	IVC, TT, CT, IVT	Secondary	Recurrence	5; 4	Dissection of OA	Progression	No	38.5
**4**	23/M	23/33	Bi	D	E	IVC, TT, CT, IVT	Secondary	Persistent	2; 1	Nil	No response	No	15
**5**	7/F	7/29	Bi	D	E	IVC, TT, CT, IVT	Secondary	Persistent	3; 3	Conjunctiva edema, Lid Edema	No response	No	21
**6**	6/M	6/13	Bi	D	D	IVC, TT	Secondary	Persistent	1; 1	Lid edema, retinal ischemia	Complete regression	Yes	13
**7**	31/M	31/31	Uni	C	Nl	IVC, TT, CT, IVT	Secondary	Persistent	3; 1	Nil	Complete regression	Yes	9
**8**	31/F	31/35	Bi	D	A	IVC, TT, IVT	Secondary	Persistent	1; 1	Nil	Partial regression	No	16
**9**	28/M	28/47	Bi	D	D	IVC, TT, CT, IVT	Secondary	Progressive	4; 2	Thrombosis of OA, lid edema, optic atrophy	Partial regression	No	25
**10**	3/M	3/19	Bi	C	C	IVC, TT, CT	Secondary	Persistent	1;1	Nil	Complete regression	Yes	14
**11**	7/M	7/37	Bi	B	D	IVC, TT, CT, IVT	Secondary	Recurrence	2; 0	Nil	-	-	33
**12**	3/F	3/21	Bi	C	B	IVC, TT, CT	Secondary	Recurrence	2; 1	Apnea, bradycardia	Complete regression	Yes	4
**13**	15/M	15/26	Bi	B	E	IVC, TT, IVT x2	Secondary	Recurrence	4; 4	Puncture site bleed	Progression	No	24
**14**	34/F	34/34	Uni	D	Nl	-	Primary	Newly diagnosed	2;2	NIL	No response	No	5

M = male; F = female; Dx: Age at diagnosis of retinoblastoma; Tx: Age at IAC treatment; Uni = unilateral; Bi = bilateral; ICRB = International Classification of Retinoblastoma; Nl = normal; IVC = Intravenous chemotherapy; TT = laser thermotherapy; CT = cryotherapy; IVT = intravitreal chemotherapy; IAC = Intra-arterial chemotherapy

*Tumor response at subsequent examination, 3–4 weeks after IAC treatment.

**Fig 2:**
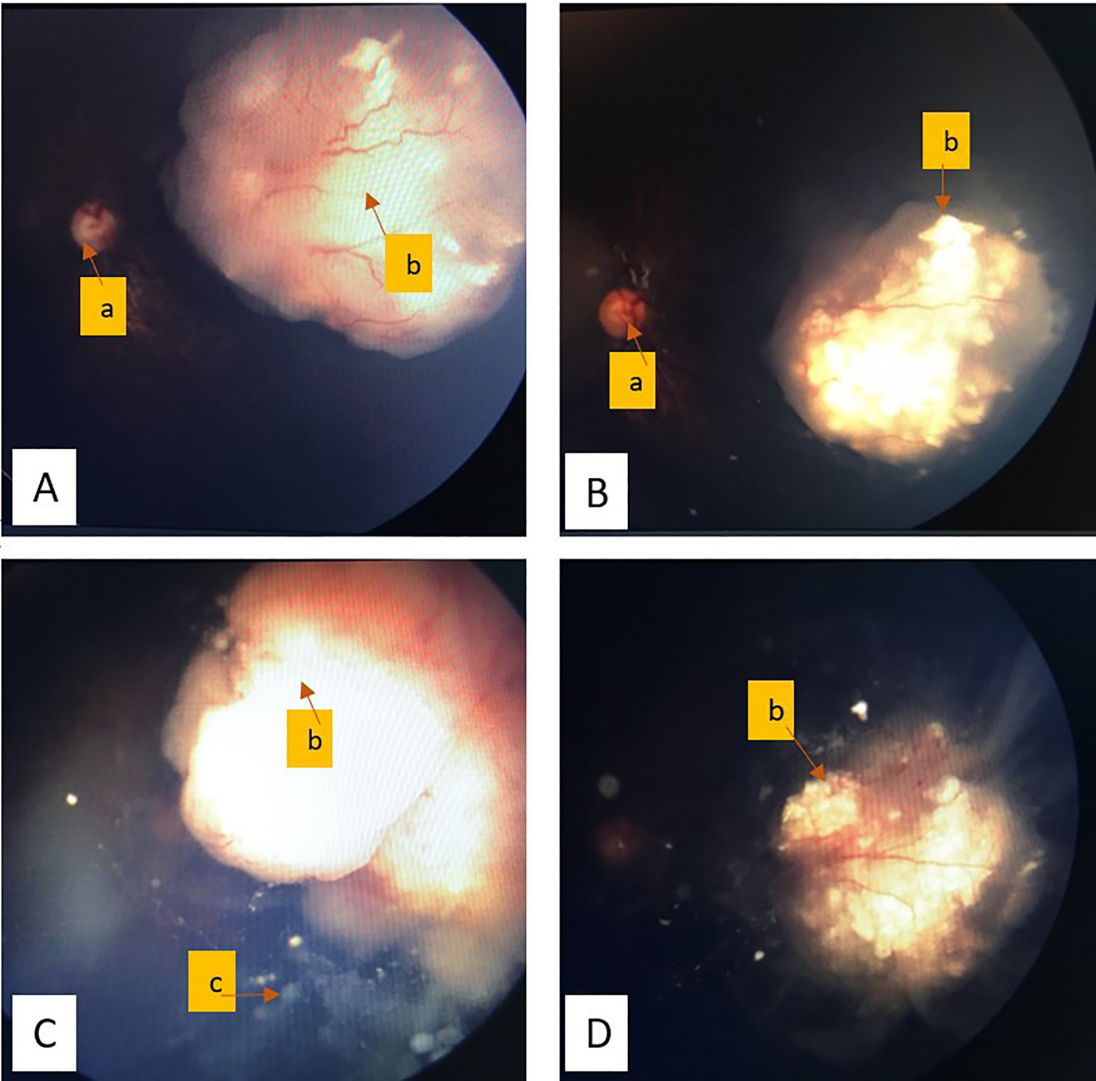
Retcam images of a retinoblastoma case showing tumor response to treatment. (A) Group B intraocular retinoblastoma at presentation, Dec 2016 (B) Tumor regression after treatment with 6 cycles of systemic chemotherapy and local treatment, Mac 2017 (C) Recurrence of tumor with vitreous & subretinal seeds 11 months later, Nov 2017 (D) Regression of tumor (Type 1: calcified remnant) 1 month after one session of intra-arterial chemotherapy (IAC), Dec 2017; a = optic disc; b = main RB tumor mass; c = vitreous seed;.

We analyzed the treatment outcome for 13 eyes that successfully received IAC (one eye that failed cannulation were excluded). Each eye received a mean number of 1.8 IAC cycle (median 1, range 1–4). All 13 eyes received melphalan as a single agent therapy. Treatment response was categorized into regression (7/13, 53%), no response/persistent (3/13, 23%) and progression (3/13, 23%). Two patients who had an initial partial regression with IAC eventually developed disease progression on follow up. They were not given further IAC because one of them failed to cannulate due to ophthalmic artery thrombosis, and another patient’s parents refused. These eyes were enucleated. After a mean follow up period of 19 months, globe salvage rate was 38%. RetCam images of one of the cases with complete regression are shown in Fig 2.

Treatment complications that were commonly reported are listed in Table 3 [11]. Adverse events of IAC were categorized into systemic, puncture site and ocular. Among 23 attempts of IAC in 14 patients, six patients (43%) experienced adverse events either intra-procedure or post-procedure. Four others had more than one complication. Most common ocular side effect in our study was lid edema and erythema (21.4%). Patient No. 12 experienced bronchospasm along with apnea and bradycardia, hence the procedure was abandoned. Bronchospasm has been reported to occur in up to 5.8% of IAC procedure, which is potentially life threatening[4]. Two of the patients’ adverse events were related to repeated IAC cannulations. Patient No. 3 had catheter-related OA dissection after four IAC sessions, causing an unsuccessful fifth attempt and the eye was enucleated; patient No. 9 developed OA occlusion after two sessions of IAC, which caused failure of the next IAC attempt. The eye also developed optic atrophy and was subsequently enucleated. Patient No. 6 developed retinal ischemia secondary to central retina artery occlusion (CRAO) after one IAC. Even though the globe was salvaged, the vision was poor.

**Table 3:** Treatment complications of 23 cannulations.

Complications	Number of eyes
**Systemic:**	
** Neutropenia**	0
** Cerebral vasoconstriction/stroke**	0
** Iodine allergy**	0
** Bronchospasm**	1
** Metastasis**	0
** Second cancer**	0
**Puncture site:**	
** Hemorrhage**	1
** Hematoma**	0
** Thromboembolism**	0
** Limb ischemia**	0
**Ocular:**	
a) *External*	
** Eyelid edema and erythema**	3
** Conjunctiva chemosis**	1
** Ptosis**	0
** Phthisis**	0
** Madarosis**	0
** Ophthalmoplegia**	0
b) *Intraocular*	
** Ophthalmic artery dissection (catheter-related)**	1
** Ophthalmic artery occlusion (thrombosis)**	1
** Retina artery occlusion (retina ischemia)**	1
** Choroidal vessel occlusion**	0
** Optic atrophy**	1
** Vitreous hemorrhage**	0
** Retinal detachment**	0

*4 patients had more than one complication

## Discussion

Retinoblastoma is potentially blinding, devastating and life-threatening if not properly treated. Its mortality rate differs by region. Early detection and advancement in treatment is the key to good prognosis of the disease. [2, 12] Current treatment options include systemic chemotherapy, enucleation, plaque radiotherapy, external-beam radiotherapy and local therapies such as laser photocoagulation, cryotherapy and thermotherapy. Meanwhile, newer treatment strategies are intravitreal injections (IVT) and intra-arterial chemotherapy (IAC). [1]

As a developing country, we started IAC for retinoblastoma in 2014. Majority of our patients presented with an advanced disease; 57% of them were of Group D and E. Most of our patients had bilateral eye retinoblastoma (79%), which echoed earlier reports of retinoblastoma presentation in Malaysia [5, 13, 14]. A local study showed that late presentation and a high rate of treatment abandonment are the major problems in Malaysia [5]. In addition, lack of public awareness and knowledge on retinoblastoma have hindered the patients from getting early medical help. Moreover, poor socioeconomic status of rural areas has also directly affected patients' accessibility to health care and adherence to treatment and follow-up. Our main reason for adopting IAC treatment was to avoid external beam radiotherapy and enucleation in our patients with advanced RB. Most of our RB patients received IAC as secondary treatment after failure of complete regression with primary treatment. Systemic intravenous chemotherapy in combination with focal therapies (laser and cryotherapy) was our first line treatment for its high efficacy and protection against metastases.

One of the factors IAC was not given as a primary mode of treatment in our center was the cost of the drug and availability of interventional radiologist. IAC is technically sophisticated and requires dedicated centers with specialized skills and instruments, which limits its availability in developing regions. Due to its steep learning curve, the same technique may not be fully reproducible between institutions, and more complications may occur under a less experienced team. [15] Using a super-selective microcatheter catheterization technique, our center's technical success rate of IAC cannulation was 71.8%, which was slightly below the success rate reported by other centers with more experience (94% - 100%). [9, 10, 15, 16] The high success rate in developed centers reflects that under expert hands and advanced facilities, this super-selective technique is very feasible with low complications; hence, our center will continue to adhere and improve our technique and skill.

Single agent IAC therapy using melphalan was used in all our patients in this study. Melphalan is widely accepted as a primary chemotherapeutic drug in IAC. Its high efficacy and short half-life make it ideal for local injection. [9] The main advantage of IAC over IVC is the ability to administer significantly higher doses of drugs directly to the tumor. This has been reported to have increased biological effect, enhanced tumor control and reduced rate of recurrence. [17] Our case series of 13 treated eyes showed tumor regression to single agent IAC in 53% of cases, which was comparable to that obtained by Shields et al. (50%). Other studies using single agent IAC such as Muen et al. and Parareda et al. reported higher tumor response rate (80% and 100% respectively) [16, 18, 19], likely due to the higher number of IAC treatment given (mean treatment range of 2.0–2.5 compared to our mean of only 1.8).

The globe salvage rate of secondary IAC in our study was 38% (5/13 globe saved) at mean of 19 months follow-up, with most cases being advanced, bilateral, refractory retinoblastoma. The globe salvage rate of other studies that also used IAC as secondary treatment in advanced/ refractory retinoblastoma ranged from 20.8% to 72.4% [16, 18, 20, 21]. A systematic review by Yoursef et al. reported a total globe salvage rate of 66%, with a salvage rate of 74% for eyes with first-line IAC and 67% for eyes with second-line IAC, with a median follow-up time of 30 months. In their review, higher globe salvage rate (86%) was achieved for eyes with less advanced RB (IIRC A-C or Reese-Ellsworth classification I-III) in comparison to eyes with more advanced RB (57%, IIRC D-E or Reese-Ellsworth classification IV-V).[4] Proposed theories for our fairly low globe salvage rate could be relative chemotherapy resistance, cells mutations from previous systemic therapy and resistance from the disease outset [22]. Even though our globe salvage rate was not overly exciting, it was still satisfactory, especially in preserving the only remaining eye in cases of advanced bilateral RB where one eye had been enucleated. The follow-up period varied in our study (range 5 to 38.5 months), a fact that could also alter the outcome and the globe salvage rate. A longer follow-up could influence our outcome as previous studies showed a decrease in globe salvage even after 32 months follow-up [20].

Primary IAC has been proven by other studies to have a better outcome compared to secondary IAC, with the improvement of success rate ranging from 10–13% in two large studies [2, 20]. Their results have helped us move towards using IAC as a primary treatment. However, ourfirst and only primarily treated eye, which was a case of Group D unilateral RB, failed (no response after two sessions of IAC and was later enucleated). Besides, literatures have shown that the combination of multiple chemotherapy agents (melphalan + topotecan/carboplatin) yielded better outcome for advanced RB after treatment failure of systemic chemotherapy, and in resistant or recurrence cases after single-agent IAC [9, 10, 23]. Therefore, our center has recently started utilizing the combination of melphalan and topotecan chemotherapy for IAC in selected refractory RB cases. This will be the next phase of our study.

We were able to demonstrate a good safety profile in our study as most adverse events we experienced were localized and transient periorbital edema or inflammation, an outcome that agrees with the systematic review by Yousef et al. They also reported vascular, ischemic and atrophic effects, as other common ocular complications with significant visual consequences, which we also encountered in three of our patients. [4]. Our IAC patients did not suffer from life threatening complications such as metastases, secondary neoplasm and death, which were reported in literatures. Our early experiences provided assurance on the safety profile of IAC and its treatment repeatability, and we do realize that a longer follow-up period may lead to further detections of events such as recurrence, metastases or death. In addition, we learned to be more thorough with patient’s pre-procedure assessment to minimize the systemic adverse events of IAC.

Our early experience in IAC has given us plenty of insights on this treatment modality as well as realizing our shortcomings. Despite the advanced RB with delayed presentation in our population, IAC can provide a good alternative to salvage the globe. Our cannulation techniques have also improved with time. Thus, we are now moving towards using a combination of therapeutic drugs (melphalan and topotecan) as well as more primary IAC treatment.

Study on retinoblastoma remains a huge challenge due to the rarity of the disease and variability in presentation and treatment response. The lack of randomized controlled trial, together with the heterogeneity across different studies in terms of presentation, treatment, tumor staging, follow-up duration and outcome, has made it difficult to fully evaluate and define the efficacy and safety of IAC in RB management. [4] Our study limitation is that this is a single center, small sample, non-comparative, retrospective study. Furthermore, our short follow-up cannot assure long lasting treatment outcome. Since IAC is a fairly new treatment modality and our study was at the initial phase of our adoption of this new technique, there is a lack of protocol to control the number of IAC sessions, time interval between IAC and adjunctive treatments. Besides, our study had no objective measurements of the tumor size and amount of retina seedings/ vitreous seedings that can better define tumor response.

## Conclusion

Our early experience has provided an added recourse in the armamentarium of RB treatment in our center, which may be useful for other developing countries that are in the early adoption of this treatment. IAC has been proven a viable treatment option to salvage globe that would conventionally require enucleation especially in bilateral RB. Prospective, systematically structured, multi-center studies with long follow up are warranted to clear the treatment uncertainties and provide more evidence on the effectiveness of IAC treatment in our population and healthcare setting. Until then, IAC should be offered selectively with thoroughly informed risk and benefits.

## Supporting information

S1 Data (XLSX)
